# Chitosan Oligosaccharides Prevent Alcohol-Induced Liver Disease by Attenuating Inflammation and Oxidative Stress

**DOI:** 10.3390/md23030134

**Published:** 2025-03-19

**Authors:** Yanglong Liu, Jiawei Sun, Qihao Yan, Bingjian Wen, Yan Bai, Qishi Che, Hua Cao, Jiao Guo, Zhengquan Su

**Affiliations:** 1 Guangdong Engineering Research Center of Natural Products and New Drugs, Guangdong Provincial University Engineering Technology Research Center of Natural Products and Drugs, Guangdong Pharmaceutical University, Guangzhou 510006, China; 18229251960@163.com (Y.L.); sunjw0724@163.com (J.S.); yanqihao980919@163.com (Q.Y.); 19860780135@163.com (B.W.); 2 Guangdong Metabolic Disease Research Center of Integrated Chinese and Western Medicine, Key Laboratory of Glucolipid Metabolic Disorder, Ministry of Education of China, Guangdong TCM Key Laboratory for Metabolic Diseases, Guangdong Pharmaceutical University, Guangzhou 510006, China; 3 School of Public Health, Guangdong Pharmaceutical University, Guangzhou 510310, China; angell_bai@163.com; 4 Guangzhou Rainhome Pharm & Tech Co., Ltd., Science City, Guangzhou 510663, China; cheqishi@rhkj.com.cn; 5 School of Chemistry and Chemical Engineering, Guangdong Pharmaceutical University, Zhongshan 528458, China; caohua@gdpu.edu.cn

**Keywords:** ALD, chitosan oligosaccharide, anti-oxidation, anti-inflammatory

## Abstract

Alcoholic liver disease (ALD) is a liver disorder resulting from excessive alcohol intake, and currently, there are no therapeutics approved by the FDA for its treatment. This study investigates the protective effects and underlying pharmacological mechanisms of two chitosan oligosaccharides, COST (MW ≤ 1000 Da) and COSM (MW ≤ 3000 Da), in mitigating alcohol-induced liver disease (ALD). In animal models, we evaluated the changes in ALD following treatment with COST and COSM. Histopathological analysis revealed that both COST and COSM interventions mitigated hepatic steatosis and inflammatory infiltration. Additionally, these compounds reduced various markers of liver injury, enhanced antioxidant enzyme levels, and significantly improved liver function. Western blot analysis demonstrated that COSM markedly decreased the expression of the hepatic metabolic enzyme CYP2E1, activated the Keap-1/Nrf-2/HO-1 pathway, and restrained the NF-κB and MAPK pathways. In an in vitro model of alcohol-induced hepatocyte L02 injury, both COST and COSM exhibited protective effects on hepatocytes, corroborating the findings from the animal studies. Collectively, in vivo and in vitro experiments confirmed that COST and COSM can reduce oxidative damage, enhance antioxidant capacity, and ameliorate steatosis and inflammatory damage in the liver, thereby significantly attenuating alcohol-induced injury. Notably, COSM exhibited slightly superior efficacy compared to COST.

## 1. Introduction

A comprehensive nationwide survey spanning from 2001 to 2016 revealed a consistent escalation in the prevalence of ALD among U.S. adults, notably marked by a significant increase in ALD cases associated with fibrosis of grade 2 or higher [1,2]. Alcohol emerges as a principal pathogenic factor in liver disease, where excessive ethanol consumption disrupts metabolic homeostasis, initially presenting as hepatic steatosis [3]. In the absence of effective regulation, this condition may progressively deteriorate, ultimately leading to liver fibrosis or even hepatocellular carcinoma, accompanied by various adverse complications [4]. The precise pathogenic mechanisms underlying ALD remain not fully elucidated. Prior studies suggest that the pathogenesis of ALD is multifaceted, involving factors such as the toxic effects of ethanol metabolites (notably acetaldehyde), oxidative stress, alterations in the gut microbiota, dysregulation of lipid metabolism, interactions between adipose tissue and the liver, impaired hepatic regeneration, and inflammatory processes [5]. Among these, the toxicity of acetaldehyde, inflammatory responses, oxidative stress, and hepatic lipid accumulation have been identified as key pathogenic mechanisms of ALD [6]. During alcohol metabolism, the generation of acetaldehyde and reactive oxygen species (ROS) induces significant oxidative stress. Elevated ROS levels compromise cellular membranes, proteins, and DNA within hepatocytes, leading to cellular dysfunction and apoptosis. Moreover, oxidative stress activates multiple inflammatory signaling pathways, thereby exacerbating hepatic inflammation. Additionally, disruptions in metabolic pathways play a crucial role in ALD pathogenesis; chronic alcohol consumption results in lipid metabolism disorders and hepatic steatosis, thereby increasing the hepatic burden and intensifying hepatocyte damage. This interplay fosters a vicious cycle that significantly accelerates the pathological progression of alcoholic liver disease [7].

Chitin is the second-only cellulose-rich polymer in the world and is a classic marine product mainly found in marine crustaceans [8]. The application of natural chitosan in the realm of marine pharmaceuticals has revealed its considerable potential as a drug delivery system. Through processes such as acid hydrolysis or enzymatic degradation, chitosan can be converted into chitosan oligosaccharides (COSs), which offer numerous advantages, including low cost, non-toxicity, water solubility, low viscosity, non-allergenic properties, enhanced gastrointestinal absorption, and the potential for long-term use. The structural configuration of COSs is characterized by the presence of N-acetylglucosamine and glucosamine units linked by β-1,4-glycosidic bonds [9]. The DD of COSs exceeds 90% [10], while its DP is less than 20, and the average MW is below 3900 Da [11]. Research has demonstrated that COSs possess a range of therapeutic properties, including antibacterial and antioxidant, anti-inflammatory [5], immunostimulatory [12], anti-obesity/anti-diabetic and wound healing activities [13,14], among others. COS regulates several critical pathways, including MAPK and NF-κB signaling. However, further research is needed to elucidate its precise molecular mechanisms [15]. CYP2E1, an essential enzyme involved in the metabolism of ethanol, catalyzes the conversion of alcohol to acetaldehyde, which subsequently elevates the levels of ROS and activates antioxidant pathways. The Keap-1/Nrf-2/HO-1 antioxidative pathway plays a vital role, as the activation of Nrf-2 leads to the upregulation of HO-1 and various other antioxidant enzymes, thereby augmenting the liver’s antioxidant capacity [16,17]. Meanwhile, acetaldehyde damages the intestinal mucosa, increasing gut permeability and leading to elevated intestinal-derived LPS and the subsequent activation of the NF-κB inflammatory pathway [18,19].

It is noteworthy that COSs with different DPs and MWs nevertheless exhibit significantly different biological anti-inflammatory antioxidant activities [20,21,22,23], however, the exact advantages and disadvantages of their biological activities are unclear. Park et al. found that COSs with a molecular weight of 1000–3000 Da had the strongest superoxide radical scavenging effect [24]. Currently, although studies have indicated that low molecular weight COSs (≤1000 Da) exhibit strong free radical scavenging capabilities, both COST and COSM demonstrate significant anti-inflammatory and antioxidant effects in the treatment of various liver diseases [25,26]. Therefore, this experiment aimed to investigate the differential protective effects of COST and COSM with varying molecular weights on alcoholic liver disease (ALD), thereby providing a foundation for the application of different molecular weight COSs in the context of ALD. Moreover, a study investigated the superoxide radical scavenging activity of five chitosan oligosaccharides (COSs) with DP ranging from 6 to 16 (molecular weight: 984–2594 Da). The results indicated that oligomers with a DP between 10 and 12 demonstrated the highest scavenging activity, implying that the optimal level of DP for COSs to effectively scavenge superoxide radicals lies within the 10–12 range [27,28,29].

**Figure 1:**
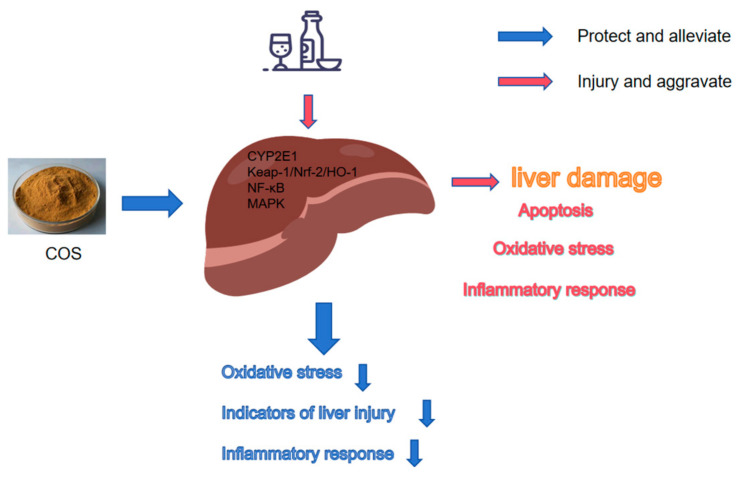
Concept map of the protective mechanism of COSs on alcohol-induced liver disease.

Oxidative damage and Inflammation are two crucial pathogenic mechanisms of alcoholic liver disease. Given the strong potent biological activities of COSs themselves, it has a high feasibility of preventing ALD, Therefore, we explored the protective effect of COSs on ALD from the cellular and animal perspectives. Figure 1 is a schematic diagram of the content of our study.

## 2. Results

### 2.1. Effects of COST and COSM on Alcohol-Induced Alt and Ast Contents in L02 Hepatocytes

**Figure 2:**
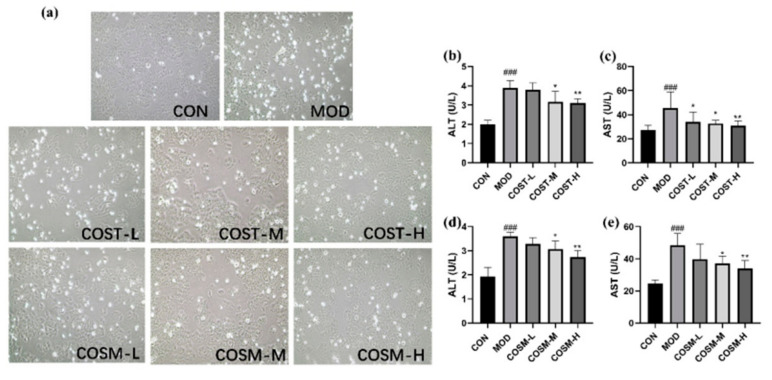
(**a**) Effects of COST and COSM on alcohol-interventional L02 hepatocyte status (200× magnification). (**b**–**e**) Effects of COST and COSM intervention on L02 liver cell ALT and AST levels (n = 6, mean ± SD). Notes: compared with the MOD group, * *p* < 0.05, ** *p* < 0.01; compared with the CON group, ### *p* < 0.001.

As can be seen from Figure 2, in the CON group, the cells exhibited a well-organized arrangement with almost no observable dead cells (bright). In contrast, the MOD group displayed a disorganized cell structure, characterized by a high presence of dead cells (bright) and altered cell morphology. Among the MOD group, the low-dose COST and COSM treatment groups demonstrated a similar cellular condition to that of the MOD group. Conversely, the medium-dose groups of both COST and COSM showed a reduction in dead cells (bright), while the high-dose groups of COST and COSM exhibited a cellular state approaching that of the CON group. In conclusion, the intervention of COSs can improve cell state, reduce cell death, and have a certain protective effect on L02 hepatocytes.

The outcomes of the assessments of L02 hepatocyte damage markers ALT and AST are illustrated in Figure 2b–e. In comparison to CON, measurements in MOD revealed significantly elevated levels of ALT and AST, attributable to alcohol-induced damage to L02 hepatocytes, which subsequently increased membrane permeability and resulted in the leakage of these enzymes into the culture medium. Within MOD group, medium and high dosage of COST treatment groups exhibited a prominent reduction in ALT levels in a dose-dependent fashion (Figure 2b). For AST, all three COST treatment groups led to a prominent decrease in enzyme levels (Figure 2c). Similarly, the COSM-M and COSM-H groups demonstrated a dose-dependent reduction in both ALT and AST levels (Figure 2d,e). These findings indicate that two different COSs have the potential to mitigate alcohol-induced injuries in L02 hepatocytes, exerting a protective effect.

### 2.2. Effects of COST and COSM on Oxidative Levels in L02 Hepatocytes

**Figure 3:**
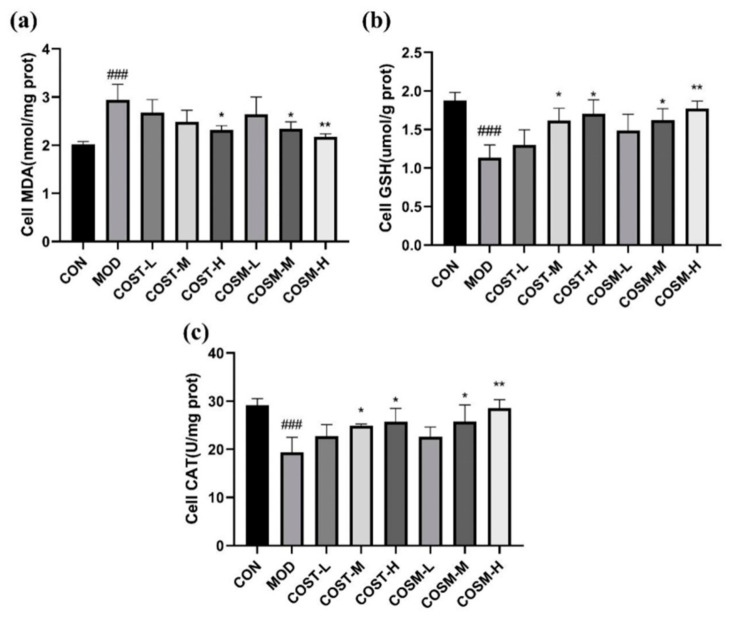
Level of oxidative stress in L02 hepatocytes: (**a**) MDA, (**b**) GSH, (**c**) CAT (*n* = 3, mean ± SD). Notes: compared with the MOD group, * *p* < 0.05, ** *p* < 0.01; compared with the CON group, ### *p* < 0.001.

MDA serves as a biomarker for lipid peroxidation and is frequently utilized to evaluate the extent of lipid peroxidation resulting from heightened oxidative stress [30]. As illustrated in Figure 3a. MDA levels in hepatocytes were apparently elevated in MOD compared to CON, suggesting that lipid peroxidation occurred in L02 cells subjected to alcohol exposure. Notably, the COST-H group effectively reduced MDA levels relative to the MOD group, while COSM also lowered MDA levels in L02 cells, with statistically significant reductions observed in both the COSM-M and COSM-H groups. These findings indicate that both COSM and COST can mitigate alcohol-induced lipid peroxidation in L02 hepatocytes, with COSM demonstrating a marginally superior effect compared to COST. Furthermore, glutathione (GSH) and catalase (CAT) are critical antioxidant enzymes involved in the metabolism of alcohol in vivo [31]. As depicted in Figure 3b,c, GSH and CAT levels were markedly diminished in MOD when contrasted with CON, indicating that alcohol exposure led to the depletion of these antioxidant enzymes in L02 hepatocytes. Conversely, both medium and high-dose treatment groups exhibited markedly enhanced levels of GSH and CAT compared to MOD (Figure 3b,c).This suggests that two different COSs can elevate the antioxidant capacity of L02 hepatocytes by boosting the activity of these essential antioxidant enzymes, with COSM revealing a slightly greater efficacy than COST at equivalent doses.

### 2.3. Effect of COS on Serum Hepatocyte Injury Markers in Mice

**Figure 4:**
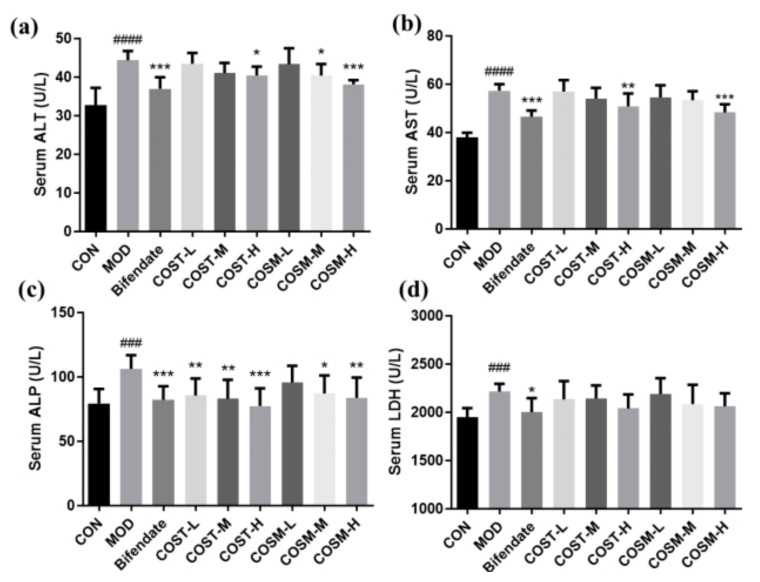
Levels of serum hepatocyte injury indicators: (**a**) ALT, (**b**) AST, (**c**) ALP, (**d**) LDH (*n* = 10, mean ± SD). Notes: compared with the MOD group, * *p* < 0.05, ** *p* < 0.01, *** *p* < 0.001; compared with the CON group, ### *p* < 0.001.

The data presented in Figure 4 indicate that serum levels of ALT, AST, ALP, and LDH were significantly elevated in MOD, signifying that excessive alcohol gavage led to liver damage in mice and successfully established an in vivo model of liver disease. Furthermore, in comparison to MOD, the COST-H group, COSM-M group, and COSM-H group demonstrated a notable reduction in ALT levels, with the COSM-H group’s effect being nearly equivalent to that of the positive control group (Figure 4a). Additionally, both the medium and high dosage of COSM treatment groups significantly decreased AST levels, with the COSM intervention showing improved outcomes over the corresponding doses of COST, and the COSM-H group exhibiting effects comparable to those of the Bifendate group (Figure 4b). Regarding serum ALP levels, all treatment groups receiving COST showed significantly lower levels than the MOD group, while the both the medium and high dosage of COSM treatment groups exhibited a dose-dependent reduction in ALP levels (Figure 4b). Regarding serum ALP levels, all treatment groups receiving COST showed significantly lower levels than the MOD group, while the COSM-M and COSM-H groups exhibited a dose-dependent reduction in ALP levels (Figure 4c). Although the changes in serum LDH levels in response to COS interventions did not reach statistical significance across the groups, a noticeable trend towards convergence was observed (Figure 4d).

In conclusion, two distinct types of COSs demonstrated a significant reduction in liver injury markers, with the most pronounced effects observed at higher dosages, approaching the efficacy of the positive control treatment. These findings suggest the potential of COSs in preventing and safeguarding the liver against alcohol-induced damage.

### 2.4. Effect of COSs on Serum Inflammatory Factors in Mice

**Figure 5:**
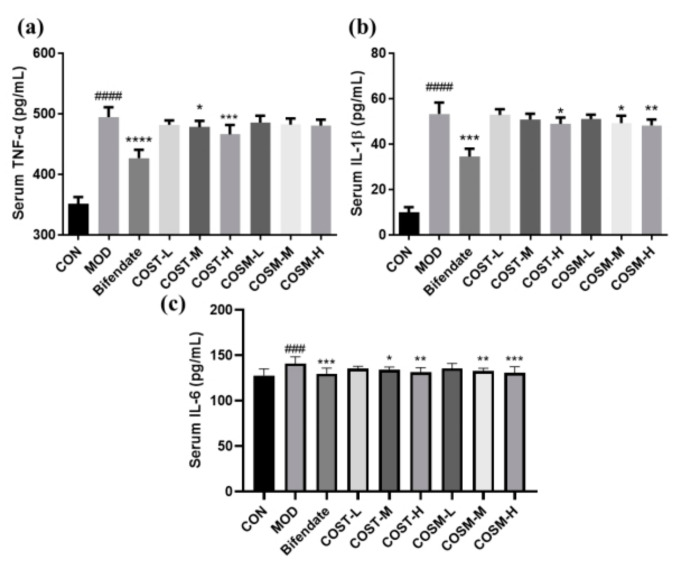
Serum inflammatory cytokines from each group of mice: (**a**) TNF-α, (**b**) IL-1β, (**c**) IL-6 (*n* = 10, mean ± SD). Notes: compared with the MOD group, * *p* < 0.05, ** *p* < 0.01, *** *p* < 0.001, **** *p* < 0.0001; compared with the CON group, ### *p* < 0.001, #### *p* < 0.0001.

Some conclusions can be analyzed from Figure 5. From the CON group, the serum TNF-α, IL-1β and IL-6 levels were obviously risen in MOD, indicating that the metabolism of excess alcohol in the liver causes an increase in inflammatory damage. In addition, the comparison between the COS intervention group and MOD showed that the medium and high dose groups of COST could obviously diminish the serum TNF-α level in mice, and there was a tendency to reduce the TNF-α level under COSM intervention but there was no statistical difference (Figure 5a). For serum IL-1β in mice, two different COS groups could significantly reduce its level at high dose, and the effect of COSM groups was dose-dependent (Figure 5b), and COST and COSM groups could obviously diminish serum IL-6, and the effect of COSM was slightly better than COST at the same dose (Figure 5c).

In conclusion, COST and COSM have alleviating and therapeutic effects on the inflammatory response caused by alcohol-induced liver disease, and in some aspects COSM plays a slightly better role than COST.

### 2.5. Effect of COS Intervention on Serum Lipid Metabolism in Mice

**Figure 6:**
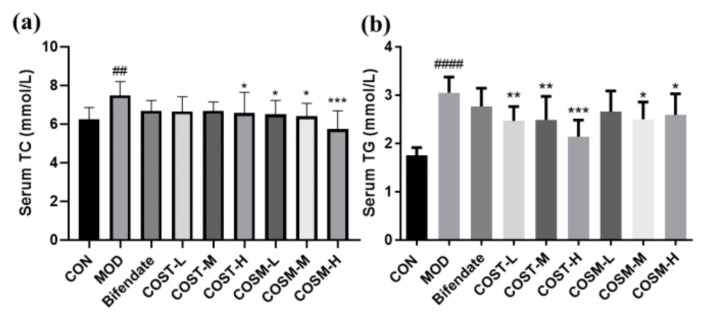
Levels of serum lipid indicators (**a**) TC and (**b**) TG in each group of mice (*n =* 10, mean ± SD). Notes: compared with the MOD group, * *p* < 0.05, ** *p* < 0.01, *** *p* < 0.001, compared with the CON group, ## *p* < 0.01, #### *p* < 0.0001.

The detection of classical serum lipid indicators TC and TG is important to understand the degree of lipid oxidation accumulation in mice, and in Figure 6, these two indicators were significantly higher in the MOD than in the CON, which is sufficient to indicate that excessive alcohol metabolism in vivo leads to increased lipid oxidation accumulation. Meanwhile, both COST-H group and COSM all dose groups could significantly reduce TC content, with the most obvious effect in the COSM-H group (Figure 6a). All COS dose groups, except the COSM low dose group, showed significantly reduced serum TG levels; however, COST performed better than COSM in terms of TG index (Figure 6b).

This suggests that two different COSs can improve lipid oxidation accumulation caused by excessive alcohol metabolism, but each has its own inhibitory effect on lipid synthesis and lipoprotein synthesis.

### 2.6. Effect of COSs on Liver Oxidative Damage Indexes in Mice

**Figure 7:**
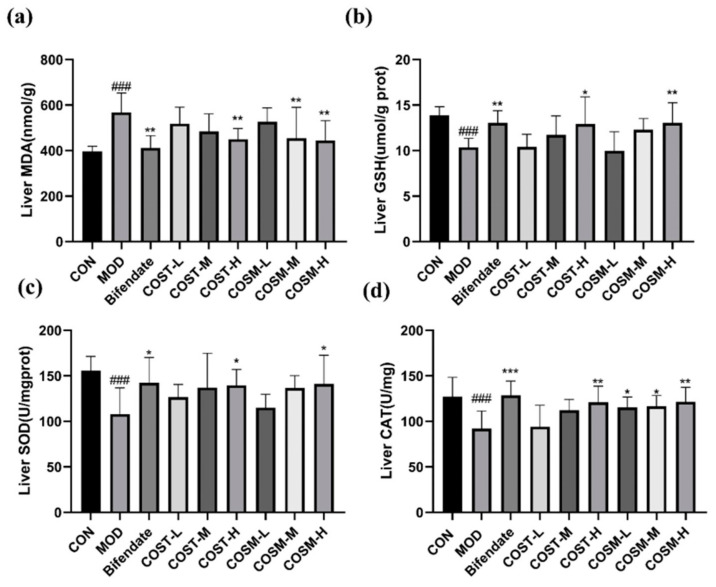
Levels of MDA (**a**), GSH (**b**), SOD (**c**) and CAT (**d**) in liver tissues of mice in each group (*n* = 10, mean ± SD). Notes: compared with the MOD group, * *p* < 0.05, ** *p* < 0.01, *** *p* < 0.001; compared with the CON group, ### *p* < 0.001.

As shown in Figure 7a, the impact of COST-H and COSM-M and H was comparable to that of Bifendate group, both of which could significantly reduce MDA levels with a dose-dependent trend. This shows that COS could reduce the damage caused by hepatic lipid peroxidation, and in addition, the most effective threshold of COSM is lower than that of COST.

In addition to the MDA index, the levels of antioxidant enzymes GSH, SOD, and CAT were obviously diminished in MOD. From CON, which can be concluded that alcohol metabolism in mice affected antioxidant capacity of the liver. From Figure 7b,c, it can be seen that the COST-H and COSM-H can obviously raise the levels of GSH and SOD to a greater extent than the Bifendate group, and it is noteworthy that for SOD, the effect of COSM-H is better than that of the COST-H group, which is corroborated by the fact that COSM is better at the same dose to increase the activity of CAT (Figure 7d).

### 2.7. Effect of COST and COSM on Pathological Sections of Mice Liver

Observing the pathological sections of the liver of mice in each group revealed some phenomena. The liver tissue in the CON group exhibited normal histological characteristics, in contrast, the hepatocytes in MOD group had poorly defined and disorganized borders, more fat vacuoles, and severe inflammatory reactions, indicating that excessive alcohol causes liver damage in mice. The fat vacuoles and inflammatory infiltration in COST-L and COSM-L were more numerous and widespread. The fat vacuoles and inflammatory infiltrates in the COST-M and COSM-M groups were slightly improved. From the low-dose group, however, when the intervention drug dose reached a high dose the inflammatory infiltrates in the liver tissues of the mice in the COST-H and COSM-H were obviously reduced, and the fat vacuoles were reduced, with the morphology of the COSM-H being the closest to that of Bifendate group.

**Figure 8:**
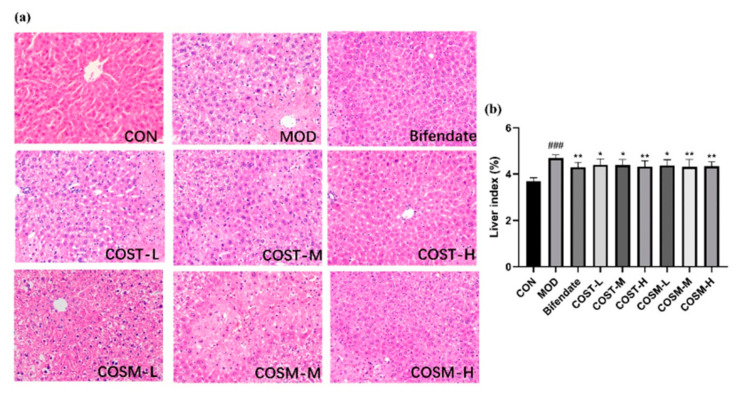
(**a**) H&E staining results of mouse liver tissue paraffin sections (200× magnification). (**b**) Comparison of liver disease indexes of mice in each group (*n* = 10, mean ± SD). Notes: compared with the MOD group, * *p* < 0.05, ** *p* < 0.01; compared with the CON group, ### *p* < 0.001.

Insights drawn from Figure 8b reveal that the liver index in MOD exhibited an obvious increase compared to that of CON. Additionally, within the MOD group, the liver indices for all doses of COSs demonstrated a marked reduction. Notably, the liver index in both high-dose groups of COSs approached those observed in the group treated with Bifendate.

### 2.8. Effect of COSM on Alcohol Metabolizing Enzyme Cyp2e1 In Vivo and In Vitro Experiments

**Figure 9:**
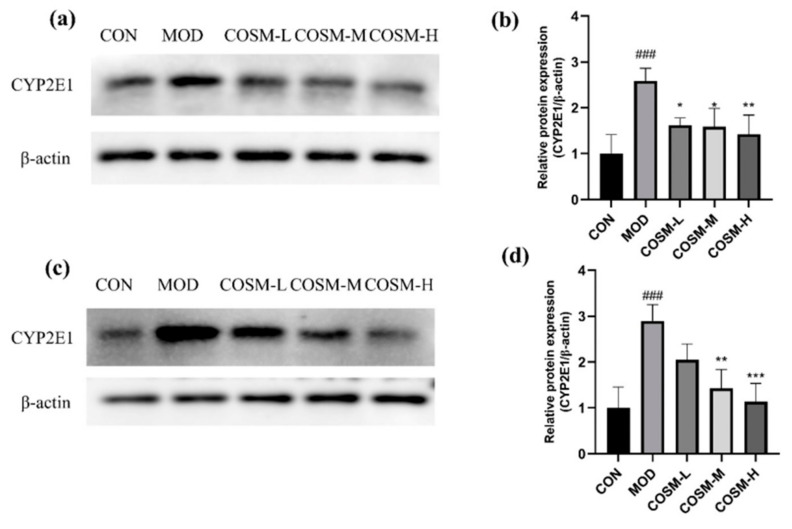
(**a**,**b**) Effects of COSM on the metabolic enzyme CYP2E1 in mouse liver (*n* = 3, means ± SD). (**c**,**d**) Effects of COSM on the metabolic enzyme CYP2E1 in L02 liver cells (*n* = 3, means ± SD). Notes: compared with the MOD group, * *p* < 0.05, ** *p* < 0.01, *** *p* < 0.001; compared with the CON group, ### *p* < 0.001.

We could detect and analyze the protein expression of CYP2E1 in mouse livers and L02 cells to understand the degree of oxidative damage caused by alcohol (Figure 9a,c). Furthermore, analysis of Figure 9b,d allows for several conclusions to be made. The expression of CYP2E1 in MOD exhibited a substantial increase from the CON. However, the COSM intervention group showed an obviously diminished expression of CYP2E1 when compared to MOD.

### 2.9. Effect of COSM on NF-κB Pathway In Vivo and In Vitro Experiments

**Figure 10:**
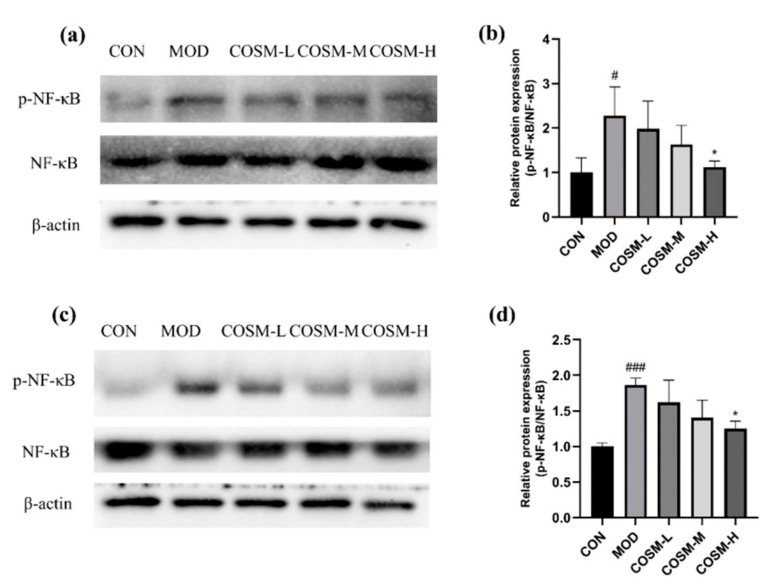
(**a**,**b**) Effects of COSM on the NF-κB pathways in mouse liver (*n* = 3, means ± SD). (**c**,**d**) Effects of COSM on the NF-κB pathways in L02 liver cells (*n* = 3, means ± SD). Notes: compared with the MOD group, * *p* < 0.05; compared with the CON group, # *p* < 0.05, ### *p* < 0.001.

As seen in Figure 10**,** in the MOD group, excessive alcohol metabolism, whether occurring in the liver or intracellularly, resulted in a notable enhancement of NF-κB phosphorylated protein expression compared to the CON group. Alcohol metabolism has the potential to activate inflammatory signaling pathways, leading to hepatic inflammatory damage. Conversely, in the MOD group, a significant reduction in NF-κB phosphorylated protein expression was observed in the COSM-H group. This finding implies that COSM may mitigate alcoholic liver disease by not only suppressing the expression of the NF-κB pathway but also by counteracting oxidative stress pathways.

### 2.10. The Effect of COSM on MAPK Signaling Pathway In Vivo and In Vitro Experiments

**Figure 11:**
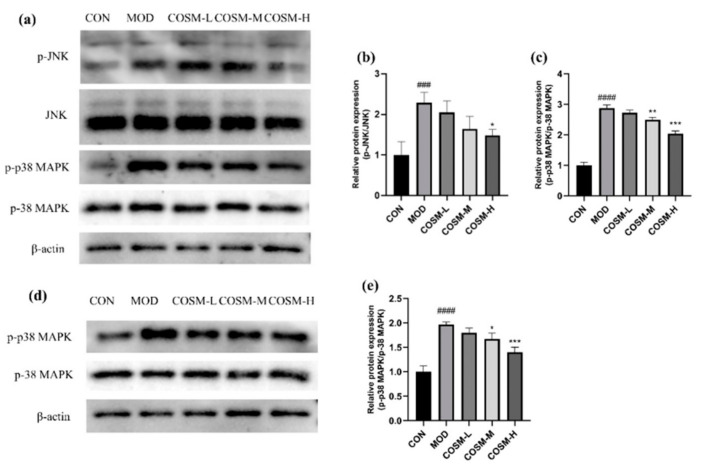
(**a**–**c**) Effects of COSM on MAPK pathways in mouse liver (*n* = 3, means ± SD). (**d**,**e**) Effects of COSM on MAPK pathways in L02 liver cells (*n* = 3, means ± SD). Notes: compared with the MOD group, * *p* < 0.05, ** *p* < 0.01, *** *p* < 0.001; compared with the CON group, ### *p* < 0.001, #### *p* < 0.0001.

During hepatic excess alcohol metabolism, Figure 11 demonstrates that the levels of phosphorylation for JNK and *p*-38 MAPK were markedly elevated in MOD in comparison to the CON, indicative of a substantial augmentation in hepatic inflammatory injury and apoptotic processes. In contrast, the phosphorylated protein expression of JNK was inhibited in the COSM-H group after the administration intervention (Figure 11a), and the expression levels of *p*-38 MAPK demonstrated a significant reduction by COSM at medium and high doses (Figure 11b).

### 2.11. Effect of COSM on the Antioxidant Pathway Keap-1/Nrf-2/HO-1 In Vivo and In Vitro Experiments

**Figure 12:**
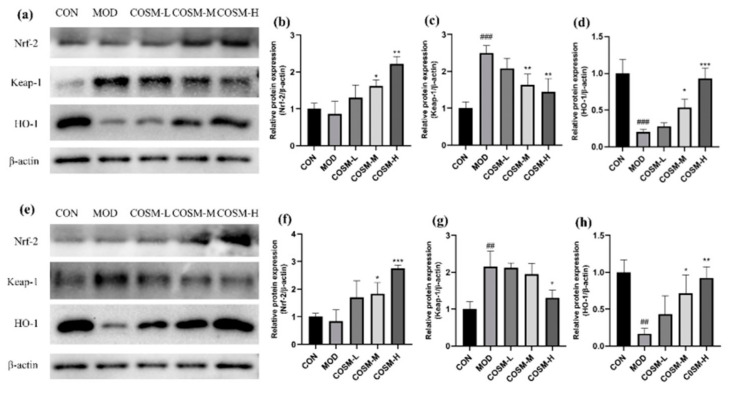
(**a**–**d**) Effects of COSM on Nrf2/the expression of proteins in the Keap1/HO-1 pathway in mouse livers. (**e**–**h**) Effects of COSM on Nrf2/the expression of proteins in Keap1/HO-1 pathway in L02 liver cells (*n* = 3, means ± SD). Notes: compared with the MOD group, * *p* < 0.05, ** *p* < 0.01, *** *p* < 0.001; compared with the CON group, ## *p* < 0.01, ### *p* < 0.001.

Oxidative pathway-related factors were examined in mouse liver tissues and L02 hepatocyte, as shown in Figure 12. From the MOD, the COSM-M and COSM-H obviously enhanced the expression of Nrf-2 (Figure 12a), but markedly suppressed the expression of Keap-1 (Figure 12b), causing more Nrf-2 to dissociate from Keap-1 and become active, transferring to the nucleus and activating antioxidant signaling, and the expression of the HO-1 was significantly increased (Figure 12c), also corroborating the improved antioxidant capacity and alleviation of oxidative damage under COSM intervention.

## 3. Discussion

Two different COSs demonstrated a significant capacity to inhibit the elevation of serum AST, ALT, ALP, and LDH in mice suffering from ALD, with the high-dose group showing effects comparable to those of the reference drug, biphenyl diester. Furthermore, two different COSs significantly diminished serum levels of TC and TG and are recognized for their ability to mitigate the accumulation of abnormal lipids. In the MOD group, intervention with COST and COSM led to a reduction in serum levels of the inflammatory cytokines TNF-α, IL-1β, and IL-6 in the mice, suggesting that both compounds may ameliorate alcoholic hepatitis, albeit with COSM exhibiting slightly superior efficacy compared to COST.

The rationale for this observation is that high molecular weight COS may enhance the activity of macrophages and lymphocytes. Research indicates that larger COS can potentiate immune cell functions and augment anti-inflammatory and immunomodulatory capacities through interaction with specific receptors, including the mannose receptor. Additionally, macromolecular COS may mitigate cellular membrane damage by forming a protective layer around the cell membrane, thereby reducing the damage rate of hepatocytes [32]. Extensive experimentation is warranted to substantiate the future potential of polysaccharides with diverse molecular weights as anti-inflammatory and anti-oxidative agents [9].

Gut-liver axis is crucial in the etiology of alcoholic liver disease. Studies indicate that COS play a significant role in regulating the gut microbiome. They facilitate the proliferation of beneficial bacteria, such as Bifidobacterium and Lactobacillus, while restrain the increase of harmful bacteria, such as Escherichia coli, thereby enhancing the stability and diversity of gut microbiome. A healthy intestinal microbiota can also facilitate hepatic metabolism and toxin excretion, mitigating the direct adverse effects of alcohol on the liver [33,34]. In the future, COSs may emerge as a novel hepatoprotective strategy in conjunction with current research trends focused on the gut-liver axis.

## 4. Materials and Methods

### 4.1. Materials

(1) Experimental Subjects: ninety specific pathogen-free (SPF) grade male Kunming mice, aged 8 weeks and weighing between 12 to 20 g, were utilized in this study, purchased from Guangdong Medical Laboratory Animal Center (Foshan, China) with animal production license number: SCXK (Guangdong) 2018-0002. (2) Chitosan oligosaccharide COST (MW ≤ 1000 Da) and chitosan oligosaccharide COSM (MW ≤ 3000 Da) sample solution: After dissolving the COST and COSM APIs in distilled water, the sample solution was obtained. COST and COSM APIs were purchased from Jinan Shandong Aokang Co., Ltd. (Shandong, China) (3) Absolute ethanol (analytical grade) was obtained from Shanghai Aladdin Biochemical Technology Co., Ltd. (Shanghai, China) (4) Samples of the positive drug Bifendate were acquired from Shanghai McLean Biochemical Technology Co., Ltd. (Shanghai, China): distilled water was used to prepare the sample solution.

### 4.2. Experimental Design In Vitro

#### 4.2.1. Cell Culture

L02 cells were obtained from the Cell Bank of the Chinese Academy of Sciences. These cells were cultured in vitro using RPMI 1640 medium (Gibco, Carlsbad, CA, USA) supplemented with 10% fetal bovine serum (HyClone, Logan, UT, USA) and 1% penicillin-streptomycin (10,000 Units/mL Penicillin, 10,000 μg/mL Streptomycin, HyClone, Logan, UT, USA), under conditions of 37 °C and 5% CO_2_. Cells were passaged or cryopreserved when they reached 70–80% confluence.

#### 4.2.2. Screening of Alcohol Modeling Concentration and Doses of COST and COSM

(1) L02 hepatocytes were inoculated in 96-well plates with 1 × 10^4^ cells per well, the 96-well plates were shaken back and forth, and the cells were observed to be evenly distributed under the microscope, transferred to the incubator for 10–12 h, and grown to the walled state. Ten experimental groups were set up with 6 replicate wells in each group, and each group was given 0, 50, 100, 150, 200, 250, 300, 350, 400, 500 mmol/L alcohol, respectively, transferred to the cell incubator and continued to incubate for 12 h, then the 96-well plates were removed and the cell survival rate of each group was determined by the CCK-8 method.

(2) The plate was seeded with 6 wells per group, and 9 groups were set up, and the administration was divided into 0, 0.25, 0.50, 1.00, 2.00, 4.00, 6.00 mg/mL COST and COSM groups. The survival rate of each group was determined by the CCK-8 method.

#### 4.2.3. Determination of the Damage and Oxidative Index in L02 Cells

(1) According to seed plate operation; a CON group, MOD group, and low-, medium- and high-dose groups of both COSs were set up, with 6 duplicate wells in each group.

(2) After screening the low, medium, and high doses of COS from the experimental results of, all groups of COS, each group was transferred to a cell culture incubator after adding corresponding concentrations of COST and COSM for 12 h. After 12 h, the MOD group and the COST and COSM intervention groups were added with the same amount of alcohol for modeling, and the CON group was added with the same amount of culture medium, and then transferred to the cell incubator for 12 h.

(3) The cellular growth condition in each experimental group was evaluated using microscopy. Subsequently, the culture medium was collected and centrifuged to isolate the supernatant. The liver injury indicators (ALT and AST) for each group were measured according to the manufacturer’s instructions for each respective assay kit, while cell viability was assessed using the CCK-8 method.

(4) After extracting the cell culture medium, the cells were digested with trypsin and homogenized in a 0.1 mol/L phosphate-buffered saline at pH 7.4. The levels of oxidative indicators (MDA, GSH, SOD, and CAT) in the homogenate were measured.

All test kits were purchased from the China Nanjing Jiancheng Biotechnology Research Institute (Nanjing, China).

### 4.3. Experimental Design In Vivo

#### 4.3.1. Animal Grouping and Administration

Mice were maintained at the Animal Center of GDPU, under the experimental unit license number SYXK (Guangdong) 2017–0125. Mice were randomly assigned to nine experimental groups, each comprising ten mice. The groups included a control group (CON), a model group (MOD), a positive drug group (Bifendate), and low, medium, and high dosage groups of COST and COSM. Each mouse was assigned an identification number and weighed.

The experiment used 5 times the recommended dose of human COS as the low dose, 10 times as the medium dose, and 20 times as the high dose. From this, dose administered to mice were COST-L: 250 mg/kg BW,COST-M: 500 mg/kg BW,COST-H:1000 mg/kg BW, and positive control (Bifendate): 150 mg/kg BW [35].

Mice in each treatment group were administered 0.1 mL of the treatment per 10 g of BW via gavage daily. In contrast, the CON and MOD were given an equivalent volume of ultra clean water. Gavage was performed once daily for a period of 30 days, during which the BW of each group of mice was recorded weekly. Following the final gavage on day 30, mice in the MOD, Bifendate, COST, and COSM groups (low, medium, and high doses) underwent a single intragastric administration of 50% ethanol at a dosage of 9 mL/kg BW to induce liver disease [36]. Mice in the CON group received the appropriate volume of distilled water and were subjected to a 16 h fasting period. Subsequently, all mice were anesthetized, and blood was harvested through ocular removal, after which the mice were euthanized via cervical dislocation. The livers were carefully excised and stored in a -80 °C freezer for future analysis.

#### 4.3.2. Serum Biochemical Analysis

Blood samples were collected using 2.0 mL EP tubes and standing for 60 min to facilitate serum separation. Subsequently, the samples were centrifuged twice at 4 °C and 3000 rpm for 15 min each to obtain serum samples [37]. The levels of liver injury markers (ALT, AST, ALP, and LDH), serum inflammatory markers (TNF-α, IL-1β, and IL-6), triglycerides (TGs), and total cholesterol (TC) were quantitatively determined. The ELISA kits for serum inflammatory markers were purchased from China Jiangsu Meimi Industrial Co., Ltd. (Taizhou, China), while the remaining kits were obtained from China Jiancheng Bioengineering Institute (Nanjing, China).

#### 4.3.3. Detection of Liver Oxidation and Antioxidant Indexes

The mouse liver tissue was removed from the −80 °C refrigerator; 0.1 g was weighed in an electronic analytical balance and transferred to a 2.0 mL sterile EP tube [38].A total of 0.9 mL of extract or physiological saline and 2 grinding beads were added to the EP tube, they were homogenized at low temperature, and then centrifuged in a low-temperature high-speed centrifuge at 4 °C and 8000 rpm for 15 min. Then, the supernatant was taken, and SOD and MDA were measured according to the kit instructions, along with GSH and CAT levels. The biological reagent kits utilized for these activity measurements were procured from China Jiancheng Biotechnology Research Institute (Nanjing, China).

#### 4.3.4. Histopathological Observation of Liver

Liver tissue of about 0.5 cm^3^ was cut into the embedding cassette and fixed with 10% formalin for 24 h. After being taken out and rinsed with pure water, it was dehydrated step by step with low to high concentration gradient ethanol, xylene, and high melting point paraffin. Immediately after dehydration, it was embedded into a tissue paraffin block with high melting point paraffin. The tissue paraffin block was pre-cooled at −20 °C for 30 min prior to being sectioned using a microtome, yielding slices of 4–5 µm in thickness. The paraffin-embedded tissue samples were then submerged in warm water to facilitate spreading and were subsequently transferred onto a pathological slide for drying. Following this, the sections underwent a series of rehydration steps, were stained with hematoxylin, followed by eosin staining, and finally dehydrated. The prepared sections were mounted with neutral gum for preservation. Ultimately, histopathological examination and photography of the sections were conducted using the bright-field setting of an upright microscope.

### 4.4. Western Blot Analysis

L02 cells and liver tissue were homogenized on ice using RIPA lysis buffer containing 1% PMSF (Meilun, Dalian, China) and allowed to sit on ice for 15 min to ensure complete lysis. The homogenate was then centrifuged at 12,000 rpm for 20 min at 4 °C, and the supernatant was collected. The protein concentration in the liver homogenate was quantified using the BCA method, and the samples were diluted to the required concentration with PBS and 5× buffer [38]. The samples were denatured in a metal bath at 98 °C for 10 min, followed by the preparation of separating and stacking gels. Approximately 10 µL of the protein sample was loaded into the wells of the electrophoresis gel. After electrophoresis, proteins were transferred to a PVDF membrane (Merck KGaA, Darmstadt, Germany). The PVDF membrane was washed three times with 1× TBST and then cut, followed by the addition of the primary antibody, which was incubated overnight. The diluted secondary antibody was applied and incubated at room temperature for 1 h. After three additional washes, chemiluminescent HRP-ECL reagent (Meilun, Dalian, China) was added for development. Images were captured and analyzed using an automated gel imaging system (ChemiDoc XRS + Bio-Rad, Hercules, CA, USA).

The antibodies utilized in this study were sourced as follows: GAPDH (10494-1-AP, rabbit anti-mouse) and horseradish peroxidase (HRP)-conjugated secondary antibody and affinity-purified IgG (H + L) (SA00001-2, goat anti-rabbit) were obtained from Proteintech Group (Wuhan, China). The remaining antibodies were procured from Abcam (Cambridge, UK).

### 4.5. Statistical Analysis

The data acquired during this experiment were analyzed and processed using SPSS version 12.0 and GraphPad Prism version 6.0 software. One-Way ANOVA was employed to assess and compare significant differences among the groups. A *p*-value < 0.05 was considered statistically significant. The results of the experiment are presented as mean ± standard deviation (SD).

## 5. Conclusions

Through research conducted using in vitro and in vivo models, we conclude that chitosan oligosaccharides, specifically COST and COSM, can protect the liver from alcohol-induced damage. Among these, COSM was identified as exhibiting superior anti-inflammatory and antioxidant properties, prompting its selection for further investigation into its pharmacological mechanisms. Our findings indicate that COSs can mitigate ALD by enhancing hepatocyte protection, improving lipid metabolism, boosting the liver’s antioxidant capacity, and reducing hepatic inflammation and apoptosis. The research establishes a theoretical framework and foundational evidence for the protective role of COS against alcoholic liver disease, highlighting its potential as a novel dietary supplement for the management of this condition.

## Data Availability

The data for the present study are available in the article.

## Abbreviations

The following abbreviations are used in this manuscript:

ALD	alcoholic liver disease
CTS	chitosan
COST	chitosan oligosaccharides (MW ≤ 1000 Da)
COSM	chitosan oligosaccharides (MW ≤ 3000 Da)
DD	degree of deacetylation
DP	degree of polymerization
MW	molecular weight
CYP2E1	cytochrome P450 proteins 2E1
Keap1	kelch-like ECH-associated protein 1
Nrf2	nuclear factor erythroid-2-related factor 2
HO-1	haem oxygenase-1
ALT	alanine aminotransferase
AST	aspartate aminotransferase
MDA	malondialdehyde
GSH	glutathione
SOD	superoxide dismutase
CAT	catalase
ALP	alkaline phosphatase
LDH	lactate dehydrogenase
TNF-α	tumor necrosis factor-α
IL-1β	interleukin-1β
IL-6	interleukin-6
TC	serum total cholesterol
TG	triglyceride
VLDL	very low density lipoprotein
ROS	reactive oxygen species
MAPK	mitogen-activated protein kinase
JNK	C-Jun N-terminal kinase
NF-κB	nuclear factor kappa-B
p38 MAPK	p38 mitogen-activated protein kinase
